# Chronic migraine management with onabotulinumtoxinA and anti-CGRP/R monoclonal antibodies in an Italian real-world setting: update on therapeutic appropriateness and an emerging role of pharmacy

**DOI:** 10.3389/fphar.2026.1868002

**Published:** 2026-07-01

**Authors:** Damiana Scuteri, Maria Diana Naturale, Martina Pagliaro, Amelia Brescia, Adele Emanuela De Francesco, Gary W. Lawrence, Giacinto Bagetta, Maria Tiziana Corasaniti

**Affiliations:** 1 Department of Health Sciences, University “Magna Grӕcia”of Catanzaro, Catanzaro, Italy; 2 Directorate-General for Animal Health, Ministry of Health, Rome, Italy; 3 Department of Pharmacy, Preclinical and Translational Pharmacology, Health and Nutritional Sciences, University of Calabria, Rende, Cosenza, Italy; 4 Pharmacy Unit, AOU Renato Dulbecco, Catanzaro, Italy; 5 Department of Biotechnology, Dublin City University, Dublin, Ireland

**Keywords:** anti-CGRP/R monoclonal antibodies, chronic migraine, migraine, onabotulinumtoxinA, pharmacist

## Abstract

Background: Migraine is a disabling neurovascular disorder that may evolve from episodic to chronic forms, often complicated by medication-overuse headache (MOH). It generates substantial healthcare expenditures and indirect societal costs. Pharmacological constraints and resistance to acute therapies contribute to the development of MOH and increase the need for preventive treatments such as onabotulinumtoxin A (BoNT/A) and monoclonal antibodies (mAbs) targeting the calcitonin gene-related peptide (CGRP) pathway. Objective: This pharmacoepidemiology retrospective study aimed to evaluate real-world prescription patterns of BoNT/A and anti-CGRP/R mAbs in an Italian regional setting and to compare current data (2023–2024) with previously observed patterns (2020–2022) before the integration of pharmacists as outpost in migraine man-agement. Methods: Anonymized data were obtained from the regional drug reimbursement and prescription database. Results: A total of 9,012 and 9,705 prescriptions were recorded in 2023 and 2024, respectively, indicating an increasing trend in the use of innovative preventive therapies. A gradual increase in the use of eptinezumab and BoNT/A appears to be associated with regional organizational measures aimed at improving access to care, adherence to clinical guidelines, and integrated management across healthcare levels. Conclusion: Therapeutic appropriateness is far from reaching the gold standard and an increasing role of pharmacists could improve clinical outcomes.

## 1 Introduction

Migraine is a complex neurological disorder associated with substantial acute disability and may progress from episodic (<15 headache days per month) to chronic forms (≥15 headache days per month of which 8 are represented by migraine days; International Classification of Headache Disorders [ICHD] −3), with an increased risk of medication-overuse headache (MOH) (Martelletti, 2015). It is the third leading cause of disability-adjusted life years (DALYs) among young females (Scuteri et al., 2025a), representing a major public health concern that impacts work productivity with significant healthcare expenditures and indirect societal costs (Charles, 2017). Acute treatment aims to abort attacks, with triptans recommended as first-line therapy for moderate-to-severe migraine when non-steroidal anti-inflammatory drugs (NSAIDs) are ineffective (Martelletti and Giamberardino, 2019). Triptans typically offer 2-h freedom from pain in 18%–50% patients, but they are contraindicated in people with hypertension due to an increased risk of coronary artery disease, stroke and peripheral vascular disease due to their vasoconstrictive properties (Scuteri and Bagetta, 2021). Inadequate migraine management has long been recognized, as highlighted by the FRAMIG 2000 study (Lucas et al., 2005). European data from the Eurolight survey indicate substantial undertreatment of migraine, with limited specialist consultation and low triptan use (6.3% of migraineurs) in Italy (Katsarava et al., 2018). French and Italian pharmacoepidemiology studies have also reported inappropriate prescribing, particularly in older patients, contributing to medication-overuse headache (MOH) (Braunstein et al., 2015; Da Cas et al., 2015). To date, data available for a wide sample of the real-world setting in Southern Italy come from a retrospective pharmacoepidemiology study aimed at evaluating triptan utilization (Scuteri et al., 2020). Real-world data further indicate substantial unmet medical needs, affecting 38.2% of triptan users (Piccinni et al., 2019). The unmet needs prompt medication overuse: limited awareness of optimal migraine management may contribute to underdiagnosis and undertreatment, paradoxically increasing the risk of medication overuse, as also suggested by Austrian data (Zebenholzer et al., 2018). The underdiagnosis and undertreatment of migraine, together with resistance to triptans and limitations to their use, lead to MOH with a consequent need for alternative preventative medications; BoNT/A or monoclonal antibodies (mAbs) directed towards either the calcitonin gene-related peptide (CGRP; eptinezumab, fremanezumab, and galcanezumab) or its canonical receptor (erenumab). BoNT/A produced by anaerobic *Clostridium botulinum* bacteria is a 900 kDa protein complex, which enters nerves and blocks their release of neurotransmitters and neuropeptides) and non-toxic accessory proteins, several having hemagglutinin activity and one non-toxic non-hemagglutinin protein (Inoue et al., 1996). Attention has been focussed on the blockade of CGRP release as a likely mechanism for the analgesic action of BoNT/A in migraine, other types of headaches, and chronic pain in other parts of the body. Nociceptive fibers originating from the trigeminal ganglion and cervical dorsal root ganglia innervate the meningeal vasculature, particularly the dura mater, and, upon activation, peripheral terminals release vasoactive neuropeptides, most notably CGRP, which induces vasodilation and pronociception. Hence, CGRP receptor antagonists and monoclonal antibodies detailed earlier inhibit the transmission of nociceptive signals originating from the dura mater. The European Headache Federation (EHF) have provided a guideline (Sacco et al., 2022) detailing moderate to high quality of evidence to recommend eptinezumab, erenumab, fremanezumab, and galcanezumab in individuals with episodic and chronic migraine. Furthermore, this guideline strongly recommended erenumab over topiramate (Sacco et al., 2022), one of the most frequently used, more effective and better tolerated among the traditional, non-specific migraine preventative small molecules (Reuter et al., 2022). A recent pharmacoepidemiology study, based on a health district consisting of 213,000 inhabitants under 60 years of age, highlighted that erenumab, galcanezumab and fremanezumab were the only preventative monoclonal antibodies prescribed (Scuteri et al., 2022). On the other hand, a meta-analysis highlighted that the treatment with BoNT/A causes fewer treatment-related adverse events (TRAEs) than topiramate (Corasaniti et al., 2023). Within this complex frame, community pharmacies are playing an increasingly important role in alleviating pressure on primary care services by delivering accessible clinical interventions for acute attacks, thus reducing the risk of chronification, helping alleviate the workload of general practitioners and decrease avoidable hospital utilization. The expansion of pharmacists’ professional responsibilities might enable the provision of more timely and individualized patient care. Within the present real-world setting, the decree n. 316 of 28 December 2023 enhanced the role of the “Chronic migraine care” project. Concurrently, in the sustainable development goals (SDG) context, these still underutilized new facilities might help to mitigate administrative and operational burdens across the wider healthcare system (BaniHani et al., 2025). Therefore, the purpose of the present retrospective pharmacoepidemiology study is to investigate the prescriptions of BoNT/A and anti-CGRP/R mAbs in the previously examined real-world setting of the southern Italy to determine a hypothesis of how this novel organization has impacted the acute treatment and, indirectly, the consequent utilization of these innovative preventive biotech therapies since 2023.

## 2 Methods

### 2.1 Aim, design and setting of the study

The real-world evidence was gathered through a retrospective study conducted in collaboration with the University Hospital Pharmacy “Renato-Dulbecco” (Pharmacy Unit, AOU Renato Dulbecco, Catanzaro, Italy). Anonymized IQVIA data were provided by the Pharmacy Unit AOU Renato Dulbecco and retrieved from the regional drug reimbursement and prescription database, including all therapeutic plans reimbursed by the National Health System (NHS). Patients suffering from episodic and chronic migraine were identified based on BoNT/A and CGRP-targeting mAbs in accordance with the European Medicine Agency (EMA) recommendations. Community pharmacists can keep under control the rate of consumption of NSAIDs and triptans, thus identifying patients potentially affected by migraine. The role of the pharmacist can include a diagnostic screening of the patients at risk of migraine and MOH for referral to the specialist. Prescriptions issued in 2020–2022 were compared with prescriptions of 2023–2024 for BoNT/A and CGRP-targeting therapies—erenumab (70/140 mg), galcanezumab (120 mg), fremanezumab (225/675 mg), and eptinezumab (100 mg). As these medications are not available over the counter, the recorded data reliably represent all reimbursed prescriptions for migraine patients experiencing more than four monthly migraine days, consistent with EMA indications. The health district covers the entire regional population, ensuring comprehensive representativeness of prescribing trends. The study was conducted in accordance with the Declaration of Helsinki.

### 2.2 Statistical analysis

Data have been extracted from the database and analyzed through Microsoft Office Excel 2010 (Microsoft, Milan, Italy). Statistical analyses have been performed using GraphPad Prism® 6.0 (GraphPad software Incorporated, San Diego, CA, USA). The results have been evaluated statistically for difference using *χ* ^2^ test for categorical variables considering *p* < 0.05 significant.

## 3 Results

Based on IQVIA data, as of 1 January 2024, in the context of the study the estimated prevalence of patients with headache disorders was 11,60%, corresponding to a total of 179.901 patients, while the estimated incidence of headache was 1,52%, corresponding to 23.573 patients. The proportion of diagnosed patients was 26,80%, corresponding to a total of 54.531 individuals. These data highlight an underestimation of migraine, in agreement with the working hypothesis. The prescription patterns of BoNT/A and anti-CGRP/R mAbs in the present real-world setting in the 2-year period 2023–2024 are distributed as it follows highlighting a total of 9.012 and 9.705 prescriptions in 2023 and 2024, respectively, as reported in Table 1.

**TABLE 1:** Real-world pattern of prescriptions in units of onabotulinumtoxin A (BoNT/A) and monoclonal antibodies against the calcitonin gene-related peptide or its receptor (anti-CGRP/R mAbs) in the two-year period 2023–2024.

Preventive therapy	2023 Units	2024 Units
Eptinezumab	6	147
Erenumab	2.308	2.282
Fremanezumab	1.327	1.562
Galcanezumab	1.703	1.829
BoNT/A	3.668	3.885
​	-	​
Total	9.012	9.705

**FIGURE 1:**
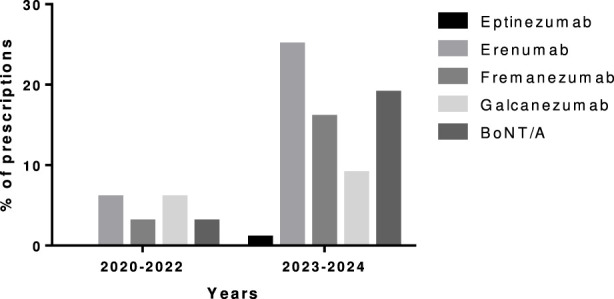
Real-world percentage of prescriptions in units of onabotulinumtoxin A (BoNT/A) and monoclonal antibodies against CGRP or its receptor (anti-CGRP/R mAbs) in comparison between the period 2020–2022 and 2023–2024. Based on descriptive results, the treatment of chronic migraine has likely improved, though not in a statistically significant manner (p = 0,2226), confirming undertreatment.

The number of prescriptions of preventive ant-CGRP biotechnological treatments (BoNT/A, and CGRP/R-targeted mAbs) for episodic and chronic migraine has been compared with data gathered within the same real-world setting in a smaller sample of a health district including 213.000 patients under 60 years of age, i.e., the population most affected by migraine, over the period 2020–2022: the data highlight an improvement in the treatment of chronic migraine, though the disease still remaining underprevented (Figure 1).

## 4 Discussion

A recent retrospective cross-sectional national health and wellness survey highlighted the need for real-world studies, to understand the unmet needs of migraine across Europe. This showed a weighted prevalence of 11.5%, with some 56% of respondents reporting disability (Coppola et al., 2025). In view of the age-standardized prevalence of migraine in Italy, calculated in the Global Burden of Disease Study 2016 as between 20.000 and 21.000 patients affected per 100.000 population (GBD 2016 Headache Collaborators, (2018)), the figure reported above points towards the underprevention of episodic and chronic migraine, which is also highlighted in the present real-world setting. Notably, the use of eptinezumab and BoNT/A has been quite limited, although it has increased over time. The use of BoNT/A has declined since the approval of CGRP-targeted treatments. Particularly, BoNT/A and eptinezumab might be the least popular treatments because their routes of administration are more complex than those of the other mAbs (Scuteri et al., 2019). In spite of the still occurring undertreatment of chronic migraine with both anti-CGRP/R mAbs and BoNT/A, an improvement, though not significant, in the use of all these drugs in the period from 2023 to 2024, can be hypothesized. For 18.717 total patients receiving these treatments, compared to 2020–2022. Although observed trends are descriptive and causality role may only be speculated, these can be explained, at least in part, by the efforts made to improve migraine patients care introduced in 2023. Likely, the purpose of this change was to ensure rapid and appropriate access to treatment, and increase availability of new therapeutic options, in accordance with clinical guidelines guaranteeing the most suitable therapy for each patient. With the Decree No. 9/2016, the Headache Network was established as an organized system structured into three levels of care. The first level includes general practitioners and community pharmacists responsible for the management of less complex cases, including low-frequency episodic migraine and chronic patients with analgesic overuse, with functions of early identification, therapeutic counselling, adherence monitoring, and referral facilitation within defined care pathways; the second level consists in outpatient neurologists, managing cases of moderate complexity, characterized by both episodic and chronic patients with low-to-moderate clinical complexity and the third level includes hospital-based Headache Centers, dedicated to the most complex and high frequency or chronic cases. These pathways are based on a multidisciplinary approach to patient management according to clinical complexity, and involve collaboration and data sharing necessary to ensure integrated and optimal patient management across all levels of care. In this real-world setting, the decree n. 316 of 28 December 2023 enhanced the role of the “Chronic migraine care” project. The results obtained support the role of community pharmacists, that emerge for the first time as frontline healthcare professionals within the regional migraine pathway, strengthening early detection of MOH, improving adherence to preventive therapies, and facilitating timely access to specialist evaluation. Ensuring high standards of healthcare delivery requires structured interdisciplinary collaboration and a solid, continuously updated knowledge of novel therapeutics, largely driven by the growing understanding of migraine pathophysiology. Within this context, pharmacists are expected to assume a relevant role in supporting evidence-based therapeutic decisions, promoting appropriate use, and contributing to the overall quality and safety of care. By operating at the primary care interface and maintaining direct, repeated contact with patients, community pharmacists are uniquely positioned to intercept inappropriate drug use early, reduce progression to chronic migraine, and mitigate the risk of MOH. Their systematic integration into the regional pathway enhances access to care, reduces diagnostic delay, and strengthens coordination between primary and specialist levels. In parallel, hospital pharmacists ensure governance of high-cost innovative therapies through validation of therapeutic plans, verification of eligibility criteria in accordance with EMA indications and national reimbursement rules, monitoring of prescription appropriateness, and evaluation of utilization trends and budget impact. This dual pharmaceutical oversight—territorial and hospital-based—reinforces regulatory compliance and sustainability of the National Health Service. Moreover, in order to guarantee efficient use of healthcare resources and improve the quality of patient care, appropriate prescribing practices are crucial. A noteworthy issue is represented by non-response and MOH. Within this frame, combination therapies are gaining much interest if able to avoid increased toxicity. Evidence from clinical trials is still lacking, but in the present real-world setting a study is ongoing assessing the efficacy of the combination of BoNT/A with eptinezumab, erenumab or atogepant, a CGRP/R antagonist known for its efficacy in non-response (Scuteri et al., 2025b). Also, this can be fundamental to reduce the risk of MOH. Overall, the structured inclusion of community pharmacists within the Headache Network represents a significant organizational innovation, contributing to improved appropriateness, optimized resource allocation, and strengthened multidisciplinary management of chronic migraine. Future, deepened studies are needed to ascertain the role of pharmacist integration in the observed increase in prescriptions of preventive medications, that actually may also be influenced by expanded availability of mAbs nationwide and patient compliance trends.

## Data Availability

The original contributions presented in the study are included in the article/supplementary material, further inquiries can be directed to the corresponding author.
